# Estimating the Survival Impact of Curative-Intent Liver Therapies for Colorectal Cancer Liver Metastases

**DOI:** 10.1245/s10434-025-17486-4

**Published:** 2025-05-22

**Authors:** Vasileios Tsagkalidis, Elizabeth A. Handorf, Benjamin A. Bates, David G. Brauer, Patrick M. Boland, Charu Verma, Mariam F. Eskander, Miral S. Grandhi, Haejin In, Hari S. Iyer, Timothy J. Kennedy, Russell C. Langan, Jason C. Maggi, Henry A. Pitt, Elisa V. Bandera, Alexander A. Parikh, Brett L. Ecker

**Affiliations:** 1 Division of Surgical Oncology, Banner MD Anderson Cancer Center, Gilbert, AZ USA; 2 Department of Biostatistics and Epidemiology, Rutgers School of Public Health, Piscataway, NJ USA; 3 Institute for Health, Health Care Policy and Aging Research, New Brunswick, NJ USA; 4 Rutgers Robert Wood Johnson Medical School, New Brunswick, NJ USA; 5 Division of Surgical Oncology, Department of Surgery, University of Minnesota, Minneapolis, MN USA; 6 Division of Medical Oncology, Rutgers Cancer Institute, New Brunswick, NJ USA; 7 Division of Surgical Oncology, Rutgers Cancer Institute, New Brunswick, NJ USA; 8 Section of Cancer Epidemiology and Health Outcomes, Rutgers Cancer Institute, New Brunswick, NJ USA; 9 Cooperman Barnabas Medical Center, Livingston, NJ USA; 10 Division of Surgical Oncology, University of Texas Health Science Center San Antonio MD Anderson Cancer Center, San Antonio, TX USA

**Keywords:** Cancer, Colorectal, Difference-in-differences, Liver, Medicare, Metastases, SEER, Survival

## Abstract

**Background:**

For patients with colorectal cancer liver metastasis (CRCLM), curative-intent liver therapies are associated with improved survival. However, randomized data are lacking, and individual-level retrospective data are limited by selection bias. We aimed to quantify the survival impact of these therapies by analyzing how regional variation in liver therapy rates influence survival outcomes within health service areas (HSA).

**Patients and Methods:**

The Surveillance, Epidemiology, and End Results (SEER)-Medicare database (2000–2020) was analyzed for patients aged 66–85 years with liver-isolated CRCLM who underwent curative-intent liver therapy (i.e., hepatectomy, ablation, or transplantation). Rates of resection within HSAs for each year were calculated. A comparison of observed versus expected survival was performed between two eras (era 1: 2002–2004; era 2: 2013–2015) to quantify the impact of changing rates of curative-intent liver therapy on HSA-level survival.

**Results:**

A total of 34,781 patients across 163 HSAs were included. Most patients had synchronous CRCLM (65.8%) and received chemotherapy (63.1%); a minority (10.9%) underwent curative-intent liver therapy (hepatectomy, 74.3%; ablation alone, 24.9%; transplantation 0.8%). A total of 56 (34%) HSAs had the rate of curative-intent liver therapy increase (≥ 5%) across eras, 58 (36%) HSAs had the rate remain constant, and 49 (30%) HSAs experienced a rate decrease (≥ 5%). Each 5% increase in the rate of curative-intent liver therapy was associated with a 1.2% (95% CI 0.4–2.0%) increase in the risk-adjusted survival rate (*p* = 0.003).

**Conclusions:**

HSA variation in rates of curative-intent therapy for CRCLM was associated with population-level changes in survival. These data quantify the expected improvements associated with efforts to increase patient access to curative-intent therapies for CRCLM.

**Supplementary Information:**

The online version contains supplementary material available at 10.1245/s10434-025-17486-4.

Colorectal cancer is the second leading cause of cancer death in the USA.^1^ Notably, colorectal cancer has remained a leading cause of cancer death for more than four decades despite a robust national screening program for detection of early stage disease. This fact suggests that an important need persists to improve the management of advanced stage disease.^2,3^ For patients with advanced disease, the most common site of metastatic spread is the liver, where colorectal liver metastases (CRCLM) develop in more than half of patients and account for two-thirds of deaths.^4,5^ Thus, improved management of patients with liver metastases is likely to improve population-level survival.

When feasible, curative-intent resection of CRCLM is considered the standard of care and can be associated with excellent long-term survival.^6^ However, surgery and systemic chemotherapy have never been directly compared in randomized studies. While advances in systemic therapies are an important contributor to improved CRCLM survival in contemporary oncology practice, the observed rate of “cure” (i.e., durable complete response) to systemic chemotherapy is exceedingly rare (< 1%).^7^ In contrast, 20–30% of patients selected for curative-intent hepatectomy will achieve long-term survival.^8,9^ Although these retrospective comparisons likely suffer from selection bias, they demonstrate that long-term survival is possible in a select group of patients with liver metastases and is increasingly observed among resection patients.^10^ An aggressive local strategy is further supported by the results of the EORTC 40004 phase II study, which demonstrated that radiofrequency ablation (with or without resection) improved progression-free survival for patients with unresectable CRCLM.^11,12^

Due to the lack of phase III randomized studies evaluating the current standard of care, alternative methods are needed to precisely quantify the impact of surgical therapies on survival for CRCLM. Area-level analyses can be used to estimate the effect of an intervention when random assignment is not feasible. In particular, temporal variations in the rate of curative-intent liver therapies can be leveraged to assess whether such changes were associated with improved overall survival. This method allows each area to serve as its own control, while adjusting for observed changes in the patient population over time. The hypothesis of this analysis was that rates of curative-intent treatment of CRCLM have temporally varied in health service areas (HSAs), and that population-level changes in rates of surgical resection can be used to estimate the effect of liver therapies on patient survival. Given that approximately one-fifth of resection patients demonstrate long-term survival, each 5% rise in curative-intent interventions would lead to a 1% increase in population-level survival. Hence, modest increases in access to these interventions across a population can yield a measurable impact on regional CRCLM survival.

## Patients and Methods

### Data Source and Patient Selection

This study consisted of a secondary analysis of data from the linked Surveillance, Epidemiology, and End Results (SEER)-Medicare database (2000–2020). Adult patients aged 66–85 years with colorectal adenocarcinoma were identified in the SEER registry by International Classification of Diseases for Oncology (ICD-O-3) topography, behavior, and histology codes, with exclusion of non-adenocarcinoma histologies (Supplementary Table 1). Identification of patients with CRCLM was accomplished using a validated algorithm requiring at least two Medicare claims for secondary malignant neoplasm of liver.^13^ Similarly, patients with extrahepatic disease were identified using site-specific Medicare claims. Patients with a diagnosis of extrahepatic metastases prior to curative-intent liver therapy, or—in the absence of such therapy—within 12 months of diagnosis of CRCLM, were excluded. The study cohort included only patients continuously enrolled in both Medicare Parts A and B for at least 12 months before diagnosis of CRCLM and were not enrolled in a managed care plan during the study period.

The date of initial diagnosis of CRCLM was considered study start time. Synchronous CRCLM was defined by a claim of liver metastasis within 1 year of CRC diagnosis, while metachronous CRCLM was defined by a claim of liver metastasis > 1 year of CRC diagnosis.

### Study Variables

The primary outcome was rate of curative-intent liver therapy, which was defined by the performance of hepatectomy, hepatic ablation and/or liver transplantation (Supplementary Table 1). Ablation was included given its equivalency in the recently presented COLLISION trial.^14^ When both hepatectomy and ablation claims were present, a window of 90 days between the two claims was used to distinguish resection with or without concurrent ablation.

Rates of curative-intent liver therapy were calculated for health service areas (HSAs), which represent local healthcare markets for inpatient care.^15^ Differences greater than 5% between time eras were defined a priori as a clinically meaningful difference. Annual residential zip code was used to determine a four-tiered categorization (urban, large rural, small rural, and isolated rural) according to the University of Washington’s categorization.^16^

### Statistical Analysis

Descriptive statistics were reported as frequencies for categorical variables and median (interquartile range [IQR]) for continuous variables. Pearson’s *χ*^2^ and Wilcoxon rank-sum test were used to analyze categorical and continuous variables, respectively. Annual rate of curative-intent liver therapy was calculated, and joinpoint regression analysis was used to model changes in annual rates.^17^ Cox proportional hazards regression model was used at the patient level to evaluate the association between overall survival and clinical variables. Patient characteristics (i.e., patient age, sex, race/ethnicity, marital status, National Cancer Institute [NCI] comorbidity index, rural urban community area [RUCA] population density, Yost index [quintiles assigned by year of diagnosis]), disease characteristics (i.e., primary tumor site, T-stage, N-stage, primary tumor grade, carcinoembryonic antigen [CEA] level at diagnosis, synchronous liver metastases [defined as interval between primary and CRCLM diagnosis < 12 months], resection of primary tumor, use of systemic chemotherapy) and hospital characteristics (i.e., medical school affiliation, bed size, Commission on Cancer [COC]-accreditation status and NCI designation) were recorded. Hospital characteristics corresponded to the hospital linked to the provider with the greatest number of claims for the patient for the first 12 months from CRCLM diagnosis. Hazard ratios and 95% CI were reported to quantify the effect of each variable on survival.

An analysis of changes in observed versus expected 1-year overall survival at the HSA-level was used to evaluate the impact of changing rates of curative-intent liver therapy on area-level outcomes. Two study eras of equal length (era 1: 2002–2004; era 2: 2013–2015) were analyzed. The overall resection rate in the period of 2002–2015 was relatively stable, as identified in joinpoint regression analysis. Prior to 2002, the rate of curative-intent therapy was rapidly changing. Following 2015, changes in ICD-9/ICD-10 coding made later periods not directly comparable. For each era, the HSA-level rate of liver surgery was calculated as well as the difference in the observed minus the expected survival between two study eras. Observed survival was the Kaplan–Meier estimate of the HSA’s 1-year survival; expected survival was the HSA’s average predicted 1-year survival, derived from a Cox regression model including patient, disease, and hospital characteristics detailed above, fit in patients who did not receive liver resection. Then, at the HSA level, changes in observed minus expected survival were associated with changes in liver resection rate using a linear regression model. *p*-Values ≤ 0.05 were considered statistically significant; all tests were two-sided. Analyses were carried out using R version 4.4.0.

## Results

### Descriptive Characteristics

A total of 79,239 patients with colorectal cancer liver metastases were identified. Following exclusion of patients with unknown follow-up (*n* = 950) outside the age range of 66–85 years (*n* = 16,302), or with extrahepatic disease (*n* = 27,206), the final cohort consisted of 34,781 patients. The clinical and demographic characteristics of the study cohort are summarized in Table 1. The majority of patients presented with synchronous liver metastases (*n* = 22,886; 65.8%) and only a minority (*n* = 3805; 10.9%) underwent curative-intent treatment of CRCLM. Initial curative-intent therapies included resection (*n* = 2826; 74.3%), with (*n* = 227; 8.0%) or without (*n* = 2599; 92.0%) ablation. Ablation alone was used in 949 patients (24.9%). Transplantation was rarely utilized (*n* = 30; 0.8%) in this elderly cohort. Among patients who underwent curative-intent liver therapy, the vast majority received systemic chemotherapy (*n* = 2946; 77.4%), and curative local therapy was performed within 6 months of diagnosis (*n* = 3006; 79.0%). When curative therapy for CRCLM was used, most patients underwent a single treatment (*n* = 3509; 92.2%), with infrequent use of repeat resection and/or ablation.

**Table 1:** Clinicodemographic characteristics of the overall cohort (*n* = 34,781)

	Total
Age, years (median; IQR)	76 (71–81)
Sex, female (%)	16,001 (46.0%)
Race/ethnicity	
Non-Hispanic white	29,297 (78.5%)
Non-Hispanic Black	3772 (10.8%)
Non-Hispanic Asian/Pacific islander	1424 (4.1%)
Non-Hispanic Native American	120 (0.3%)
Hispanic	2121 (6.1%)
Unknown	37 (0.1%)
Marital status	
Partnered	13,235 (38.1%)
Unpartnered	10,680 (30.7%)
Not reported	10,866 (31.2%)
NCI comorbidity index	
< 1	27,923 (80.3%)
1+	5321 (15.3%)
Unknown	1537 (4.4%)
RUCA population density	
Urban-focused	27,225 (78.3%)
Large rural city	2814 (8.1%)
Small rural town	1539 (4.4%)
Isolated small rural town	1063 (3.1%)
Unknown	2140 (6.2%)
Yost	
1st (lowest SES)	4489 (12.9%)
2nd	4673 (13.4%)
3rd	4938 (14.2%)
4th	5399 (15.5%)
5th (highest SES)	5726 (16.5%)
Unknown	9556 (27.5%)
Primary site	
Right-sided	12,768 (36.7%)
Left-sided	12,404 (35.7%)
Rectum	5444 (15.7%)
Overlapping	4165 (12.0%)
T-classification	
T1	1944 (5.6%)
T2	1493 (4.3%)
T3	10,283 (29.6%)
T4	3962 (11.4%)
TX	17,099 (49.2%)
N-classification	
N-negative	9635 (27.7%)
N-positive	9764 (28.1%)
NX	15,382 (44.2%)
Grade	
G1	1868 (5.4%)
G2	19,733 (56.7%)
G3	7226 (20.8%)
GX	5954 (17.1%)
CEA	
Normal	3035 (8.7%)
Elevated	7610 (21.9%)
Unknown	24,136 (69.4%)
Synchronous CRCLM	22,886 (65.8%)
Primary tumor resection	26,586 (76.4%)
Systemic chemotherapy	21,931 (63.1%)
Hospital medical school affiliation	16,835 (48.4%)
Hospital CoC accreditation	17,195 (49.4%)
Hospital NCI designation	3269 (9.4%)

*COC* Commission on Cancer, *CRCLM* colorectal cancer liver metastasis, *NCI* National Cancer Institute, *RUCA* rural urban community area, *SES* socioeconomic status

### Annual Rates of Curative-Intent Liver Therapy

**Fig. 1:**
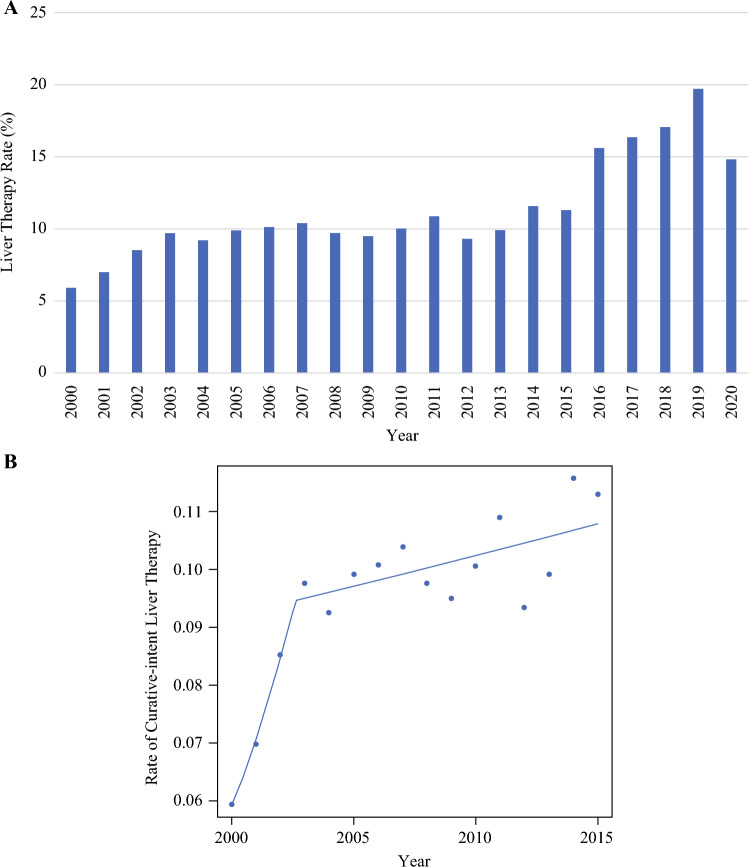
**A** Annual rates of curative-intent livery therapy for CRCLM, shown as percentage in each year; **B** joinpoint regression analysis with identification of significant change in therapy rates in the year 2002

The rate of curative-intent therapy was analyzed by year of CRCLM diagnosis (Fig. 1A). The rate increased from 5.9 to 8.5% during the years 2000–2002, with a plateau observed in the annual rate (mean rate 10%) during the years of 2002–2015. Joinpoint regression analysis confirmed a significant difference in liver therapy rates corresponding with the year 2002 (*p* < 0.001; Fig. 1B). Corresponding with a change from ICD-9 to ICD-10 coding at the end of 2015, the rates of hepatectomy changed in 2016 and ranged from 15.6% to 19.7% for the years 2016–2019. In the year 2020, a 14.8% rate of curative-intent therapy was observed.

### Impact of Changing Rates of Curative-Intent Liver Therapy

At a median follow-up of 137.0 (IQR 68.0–198.0) months, median OS in the overall cohort was 7.0 (IQR 2.0–21.0) months. Curative-intent liver therapy was associated with significantly improved survival (HR 0.53, 95% CI 0.51–0.56, *p* < 0.001) after adjusting for patient-, tumor-, and hospital-related variables (Table 2).

**Table 2:** Multivariable Cox regression analysis for overall survival

	HR (95% CI)	*p*-Value
Liver therapy	0.53 (0.51–0.56)	< 0.001
Age, years	1.02 (1.01–1.02)	< 0.001
Sex		
Female	Ref.	
Male	1.04 (1.01–1.07)	0.005
Race/ethnicity		
Non-Hispanic white	Ref.	
Non-Hispanic Black	1.04 (1.00–1.08)	0.025
Non-Hispanic Asian/Pacific islander	0.88 (0.83–0.93)	< 0.001
Non-Hispanic Native American	0.84 (0.64–1.11)	0.218
Hispanic	0.97 (0.92–1.02)	0.258
Marital status		
Partnered	Ref	
Unpartnered	1.07 (1.03-1.11)	<0.001
Not reported	0.87 (0.84-0.91)	<0.001
NCI comorbidity index		
< 1	Ref	
≥ 1	1.14 (1.11-1.18)	<0.001
Yost index		
1 (lowest SES)	Ref.	
2	0.94 (0.90–0.99)	0.003
3	0.93 (0.90–0.97)	0.001
4	0.91 (0.87–0.95)	< 0.001
5 (highest SES)	0.87 (0.84–0.91)	< 0.001
Unknown	0.96 (0.92–1.02)	0.170
Primary site		
Left-sided	Ref.	
Right-sided	1.09 (1.06–1.12)	< 0.001
Rectum	1.05 (0.99–1.10)	0.057
Overlapping	1.11 (1.07–1.16)	< 0.001
T-classification		
T1	Ref.	
T2	0.87 (0.81–0.94)	< 0.001
T3	0.96 (0.91–1.03)	0.244
T4	1.12 (1.05–1.20)	< 0.001
TX	1.10 (1.03–1.18)	0.004
N-classification		
N-positive	Ref.	
N-negative	0.83 (0.80–0.86)	< 0.001
NX	0.95 (0.91–0.99)	0.007
Grade		
G1	Ref.	
G2	1.05 (0.98–1.13)	0.132
G3	1.31 (1.11–1.41)	< 0.001
GX	1.21 (1.12–1.30)	< 0.001
CEA		
Normal	Ref.	
Elevated	1.20 (1.15–1.26)	< 0.001
Primary tumor resection		
No	Ref	
Yes	0.64 (0.61–0.66)	< 0.001
Systemic chemotherapy		
No	Ref.	
Yes	0.59 (0.56–0.62)	< 0.001
Liver metastasis		
Metachronous	Ref.	
Synchronous	1.19 (1.15–1.24)	< 0.001
Medical school affiliation		
No affiliation	Ref.	
Major	0.97 (0.92–1.02)	0.205
Limited	0.97 (0.94–1.01)	0.160
Graduate	0.94 (0.89–0.99)	0.041
Hospital size		
≤ 100 beds	Ref.	
101–200 beds	1.03 (0.97–1.09)	0.299
201–500 beds	1.02 (0.96–1.07)	0.559
> 500 beds	0.98 (0.920–1.06)	0.462
Hospital CoC-accreditation		
No	Ref.	
Yes	1.01 (0.97–1.04)	0.760
Hospital NCI designation		
None	Ref.	
Comprehensive	0.78 (0.70–0.87)	< 0.001
Clinical	0.85 (0.78–0.93)	< 0.001
Year of CRCLM diagnosis	0.99 (0.98–0.99)	< 0.001

*COC* Commission on Cancer, *CRCLM* colorectal cancer liver metastasis, *NCI* National Cancer Institute, *SES* socioeconomic status

Given the observed plateau in the national rates of curative-intent liver therapy during the years 2002–2015, two eras were defined within this range of equal length: era 1 (2002–2004) and era 2 (2013–2015). A total of 220 unique HSAs containing at least one study patient were identified. After exclusion of 57 HSAs with less than 1 patient during either of the periods of interest, a final study cohort of 163 HSAs comprising 10,585 patients (era 1: *n* = 6195; era 2: *n* = 4390) was analyzed (Supplementary Table 2).

**Fig. 2:**
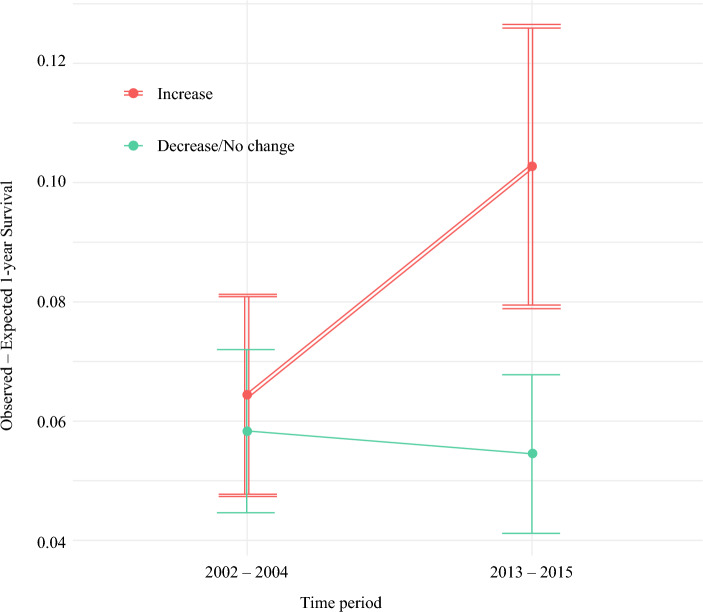
Changes in observed versus expected survival rates across eras, stratified by health service areas where the rate of curative-intent liver therapy increased (red double line) or decreased/remained unchanged (blue single line)

In this cohort, the observed 1-year survival during era 1 was 36.8% and increased to 37.7% in Era 2 (*p* = 0.002). A total of 56 (34%) HSAs had the rate of curative-intent liver therapy increase by more than 5% between eras, 58 (36%) HSAs had the rate remain constant, and 49 (30%) HSAs had the rate decrease by more than 5%. By comparison of observed versus expected 1-year survival, those HSA where the rate of curative-intent liver therapy increased, the associated increase in survival exceeded the expected changes on the basis of patient, disease, and hospital characteristics as well as general management in a more contemporary era (*p* = 0.003) (Fig. 2). An increase in the curative-intent liver therapy rates of 5% of a given HSA was associated with a 1.2% (95% CI 0.4–2.0%) increase in the HSA 1-year survival rate (over the expected rate) (Supplementary Fig. 1). Moreover, when limited to the subset of patients who received any cancer-directed therapies (i.e., systemic chemotherapy, curative-intent liver therapy, or both), an increase in HSA rates of curative-intent liver therapy remained associated with improved survival (*p* = 0.006; Supplementary Fig. 2).

Increases in rates of curative-intent liver therapy may occur in conjunction with improved use of systemic therapy, particularly if occurring in high-volume institutions with both surgical and medical oncology expertise. To exclude this potential source of bias, a sensitivity analysis was performed after excluding patients who underwent liver therapy. HSAs where the rate of curative-intent liver therapy increased were no longer associated with improvements in survival (*p* = 0.570; Supplementary Fig. 3).

## Discussion

Liver-directed therapies (i.e., surgical resection, ablation, and/or transplantation) are important tools to achieve cure for CRCLM, although their contemporary use and their precise impact on survival are not well defined. Herein, by analysis of a national cohort of eligible Medicare patients with liver-isolated CRCLM, changes in regional variation in liver therapy rates were leveraged to quantify their survival impact. Each 5% increase in the rate of curative-intent liver therapies was associated with a greater than 1% increase in the risk-adjusted population-level survival, and which could not be explained by other changes in the multidisciplinary management of these patients. These results highlight the crucial role of curative-intent liver therapies in improving survival outcomes for patients with CRCLM and provide quantification of the expected improvements from interventions that improve access to and completion of these therapies.

No phase III randomized controlled trials directly compare the efficacy of systemic chemotherapy versus the standard-of-cure surgical resection in patients with resectable CRCLM. The survival impact of curative-intent liver therapy is widely reported in multiple retrospective population-based studies.^5,6,18^ In this study cohort, patients undergoing liver-directed therapies (primarily hepatectomy) demonstrated improved survival (HR 0.53, 95% CI 0.51–0.56). However, these retrospective analyses are limited by the presence of a strong selection bias, immortal time bias, and unmeasurable confounders. Given these limitations, an area-level analysis is a preferred strategy to estimate causal effect from observational data. Examined at the level of health service areas, which are likely independent of unmeasured confounders such as liver tumor burden, this methodology limited selection bias and allowed for robust comparison of regional survival outcomes. Despite a constant national rate of liver-directed therapies for the years 2002–2015, regional pockets of increased use as well as decreased use were observed during this time. Using these variations as a natural experiment, differences in regional rates were exploited to assess their impact on regional risk-adjusted survival.

Nationally, several changes in use of liver therapies for CRCLM were observed. The joinpoint analysis confirmed that the year 2002 marked a significant inflection point in utilization of liver therapies. This time corresponds with previously published data from the M.D. Anderson Cancer Center and the Mayo Clinic, where a doubling in the rate of surgical resection was observed for patients with CRCLM between 2000 and 2002.^19^ Several plausible explanations exist for this sudden national change in practice. First, modern chemotherapy was introduced during this time, with publication of benefit of first-line oxaliplatin (2000), with increasing national use by 2002.^19,20^ This advancement may have changed the number of patients with technically resectable disease, by virtue of tumor downstaging, and it may have altered the numbers of patients with “biologically resectable” disease, by virtue of improved systemic control. Second, there may have been a national change in surgeon experience with liver resection. Hepatectomy for CRCLM during the period from 1950 to 1980 was associated with significant morbidity and mortality, with greater than one-third of patients experiencing postoperative liver failure and one-fifth of patients suffering postoperative death.^21,22^ However, with improved understanding of surgical anatomy and technique, several series from high-volume institutions were published in the 1990s, demonstrating operative mortality < 5%.^23^ The increased use of liver therapies in the early 2000s may have corresponded with the national spread of these modern techniques.

No changes in the national rate of liver therapies were observed in the present analysis from 2002 through 2015. During this time, targeted therapies (e.g., bevacizumab and cetuximab in 2004), which may increase the number of patients who are converted to resectable disease, were introduced.^24–28^ By 2019, the last complete year of study data prior to the coronavirus disease 2019 (COVID-19) pandemic, the national rate of curative-liver therapy reached 20%, which closely matches resectability rates observed in several single-institution series.^5,18,19,29,30^ This observation may signify that contemporary national practice may have achieved appropriate capture of patients with resectable CRCLM for curative-intent local therapies.

Several limitations warrant emphasis. First, while hepatectomy, liver ablation, and transplantation are not typically performed for palliative purposes, the success of these curative-intent therapies, such as the treatment margin, was not assessed. In addition, this analysis was limited by the lack of certain details captured in SEER, most importantly the number, distribution, and mutational status of liver tumors. While all patients with extrahepatic disease were excluded, significant variation in disease burden for liver-only CRCLM undoubtedly remains among these patients. However, an HSA-level analysis, where the distribution of liver tumors across large health regions can be assumed to be similar, overcomes the unmeasured source of bias that would otherwise be present in patient-level analysis. Third, the SEER data do not include information about metastasis occurring after initial diagnosis and provide limited information regarding site of metastasis. The algorithm utilized to identify this study cohort in Medicare claims has > 90% specificity for identifying metastatic colorectal cancer, but the limited sensitivity of this methodology likely excluded eligible study patients, potentially underestimating the true burden of disease.^13^ Fourth, the time frame of this study included both the use of ICD-9 diagnosis codes (years 2000–2015) and ICD-10 diagnosis codes (years 2016–2020), with observed differences in the rate of resection in 2016. This is likely related to improved patient capture with ICD-10 coding, as there is not a clinical reason for the increased use of hepatectomies beginning in 2016. The eras defined for the area-level analyses were limited to ICD-9 coding to avoid this potential discrepancy and further consideration of hepatectomies in more recent years is needed. Fifth, details regarding specific chemotherapy agents used were not reliably reported, limiting further analyses of the rates of liver therapy stratified by treatment regimen. Treatment regimens and chemotherapy dose-density may have varied and can contribute to biases in survival analyses at an individual patient level; however, the HSA-level analysis reduced the influence of this unmeasured confounder. Additionally, use of chemotherapy was adjusted for in survival analyses. Lastly, this study population was elderly and possibly frail, as evidenced by one-third of patients not receiving any chemotherapy. This likely contributed to the poor survival outcomes of the overall cohort, regardless of treatment strategy employed. Notably, the association between changes in regional rates of liver therapy and HSA-level survival remained when excluding patients who did not receive any cancer-directed therapies, who may have had rapidly progressing disease or life-limiting comorbidities. The impact of changes in regional rates of curative-intent therapies may be more pronounced among a younger patient cohort where the competing risk of non-cancer death is lower and tolerance for aggressive systemic therapies is greater.

## Conclusions

In this large population-based cancer registry spanning over two decades of contemporary practice, national rates of curative-intent liver therapies for patients with liver-isolated CRCLM have significantly increased. Regional variation in such therapies were observed, where an increase in the rate of liver therapies within an HSA was associated with an improvement in the region’s risk-adjusted survival. These data provide quantification of the expected survival benefit associated with efforts to improve resectability rates, while identifying potential regional disparities in curative therapies. Further investigation is needed to understand the factors contributing to the variability in liver therapy rates across different health service areas as a potential source of disparity in cancer outcomes.

## Supplementary Information

Below is the link to the electronic supplementary material.

Supplementary file1 (DOCX 21 KB)

Supplemental Figure 1. Graphical representation of the area-level analysis. Each point on the scatterplot represents an HSA. The x-axis measures the change in rates of surgery between the two time periods (Era 1: 2002-2004; Era 2: 2013-15). The y-axis measures the change in the observed – expected 1-year survival between the two time periods. The fitted regression line shows the relationship between these measures. The marginal histograms show the distribution of the changes. Supplemental Figure 2. Changes in observed vs. expected survival rates across eras, stratified by health service areas where the rate of curative-intent liver therapy increased (red double line) or decreased/remained unchanged (blue single line) for the subset of patients who received any cancer-directed therapy (e.g., chemotherapy, curative-intent liver therapy or both). Supplemental Figure 3. Changes in observed vs. expected survival rates across eras, stratified by health service areas where the rate of curative-intent liver therapy increased (red double line) or decreased/remained unchanged (blue single line) for the subset of patients who did not receive curative-intent liver therapy (PDF 1076 KB)

## Data Availability

The datasets used to conduct this study are available upon approval of a research protocol from the National Cancer Institute. Instructions for obtaining these data are available at https://healthcaredelivery.cancer.gov/seermedicare/obtain/
