# Supplementary material for: Estimating the Survival Impact of Curative-Intent Liver Therapies for Colorectal Cancer Liver Metastases
Source: Ann Surg Oncol. 2025 May 22;32(9):6263–71. doi: 10.1245/s10434-025-17486-4 (PMC12317857; doi:10.1245/s10434-025-17486-4)
Supplement: Supplementary file 1 — Supplementary file1 (DOCX 21 KB) [file 10434_2025_17486_MOESM1_ESM.docx]

**SUPPLEMENTAL TABLES**

| **Supplemental Table 1**. ICD-O-3, ICD-9/10 and CPT codes |
| --- |
| **Histology Adenocarcinoma**: 8140-8145, 8210-8211, 8213, 8220-8221, 8230, 8255, 8260-8263, 8480, 8490, 8560, 8570-8574 |
| **Primary site**: Right colon C180, C182-183, Left Colon C185-187, C199, Rectum C20, C209, Other C184, C188, C189 (Appendix Primary C181 excluded) |
| **Metastatic sites**: Liver (ICD-9 codes: 155.2, 197.7, ICD-10 codes: C22.9, C78.7), Lung (ICD-9 code: 197.0, ICD-10 codes: C78.0, C78.00, C78.01, C78.02), Bone (ICD-9 code: 198.0, ICD-10 code: C79.5), Pleura (ICD-9 code: 197.2, ICD-10 code: C78.2), Peritoneum (ICD-9 code: 197.6, ICD-10 code: C78.6), Brain (ICD-9 code: 198.3, ICD-10 codes: C79.31–C79.32), Adrenal (ICD-9 code: 198.7, ICD-10 codes: C79.70–C79.72) |
| **ICD-10 Procedure Codes**: Partial hepatectomy (0FB00ZZ, 0FB03ZZ, 0FB04ZZ, 0FT00ZZ, 0FT04ZZ), lobectomy (0FB10ZZ, 0FB13ZZ, 0FB14ZZ, 0FB20ZZ, 0FB23ZZ, 0FB24ZZ, 0FT10ZZ, 0FT14ZZ, 0FT24ZZ, 0FT20ZZ), liver ablation (0F500ZZ, 0F503ZZ, 0F504ZZ, 0F510ZZ, 0F513ZZ, 0F514ZZ, 0F520ZZ, 0F523ZZ, 0F524ZZ), liver transplantation (0FY00Z0)  **ICD-9-CM Procedure Codes**: partial hepatectomy/ wedge resection (50.22), lobectomy of liver (50.3), liver ablation (50.23, 50.24, 50.25, 50.26), liver transplantation (00.91, 00.92, 00.93, 50.59)  **CPT**: partial lobectomy (47120), trisegmentectomy (47122), total left lobectomy (47125), and total right lobectomy (47130), liver ablation (76940, 77013, 77022, 47380, 47381, 47382, 47383, 47370, 47371), liver transplantation (47133, 47135, 47140, 47141, 47142, 47143, 47144, 47145, 47146, 47147) |

| **Supplemental Table 2**. Clinicodemographic characteristics of the overall cohort, stratified by eras of diagnosis | | | | |
| --- | --- | --- | --- | --- |
|  | | **Era 1**  **N=6,195** | **Era 2**  **N=4,390** | **p-value** |
| Age, years (median; IQR) | | 77.0 (72.0-81.0) | 76.0 (71.0-81.0) | <0.001 |
| Sex, female (%) | | 2,997 (48.4%) | 1,940 (44.2%) | <0.001 |
| Race/ethnicity | Non-Hispanic White | 5,044 (81.4%) | 3,315 (75.5%) | <0.001 |
|  | Non-Hispanic Black | 620 (10.0%) | 510 (11.6%) |  |
|  | Non-Hispanic Asian/Pacific islander | 194 (3.1%) | 218 (5.0%) |  |
|  | Non-Hispanic Native American | 17 (0.3%) | 21 (0.5%) |  |
|  | Hispanic | 318 (5.1%) | 323 (7.4%) |  |
|  | Unknown | 2 (0.0%) | 3 (0.1%) |  |
| Marital status | Partnered | 2,351 (37.9%) | 1,672 (38.1%) | 0.011 |
|  | Unpartnered | 1,841 (29.7%) | 1,404 (32.0%) |  |
|  | Not reported | 2,003 (32.3%) | 1,314 (29.9%) |  |
| NCI comorbidity index | <1 | 5,310 (85.7%) | 3,367 (76.7%) | <0.001 |
|  | 1+ | 622 (10.0%) | 814 (18.5%) |  |
|  | Unknown | 263 (4.2%) | 209 (4.8%) |  |
| RUCA population density | Urban-focused | 5,110 (82.5%) | 3,429 (78.1%) | <0.001 |
|  | Large rural city | 506 (8.2%) | 357 (8.1%) |  |
|  | Small rural town | 284 (4.6%) | 213 (4.9%) |  |
|  | Isolated small rural town | 200 (3.2%) | 135 (3.1%) |  |
|  | Unknown | 95 (1.5%) | 256 (5.8%) | <0.001 |
| Primary site | Right-sided | 2,293 (37.0%) | 1,589 (36.2%) |  |
|  | Left-sided | 2,291 (37.0%) | 1,507 (34.3%) |  |
|  | Rectum | 926 (14.9%) | 718 (16.4%) |  |
|  | Overlapping | 685 (11.1%) | 576 (13.1%) |  |
| T-classification | T1 | 172 (2.8%) | 355 (8.1%) | <0.001 |
|  | T2 | 212 (3.4%) | 196 (4.5%) |  |
|  | T3 | 1,475 (23.8%) | 1354 (30.8%) |  |
|  | T4 | 461 (7.4%) | 625 (14.2%) |  |
|  | TX | 3,875 (62.6%) | 1,860 (42.4%) |  |
| N-classification | N-negative | 1,378 (22.2%) | 1,433 (32.6%) | <0.001 |
|  | N-positive | 1,404 (22.7%) | 1,525 (34.7%) |  |
|  | NX | 3,413 (55.1%) | 1,432 (32.6%) |  |
| Grade | G1 | 334 (5.4%) | 232 (5.3%) | <0.001 |
|  | G2 | 3,568 (57.6%) | 2,493 (56.8%) |  |
|  | G3 | 1,487 (24.0%) | 865 (19.7%) |  |
|  | GX | 806 (13.0%) | 900 (18.2%) |  |
| CEA | Normal | 90 (1.5%) | 562 (12.8%) | <0.001 |
|  | Elevated | 412 (6.7%) | 1,350 (30.8%) |  |
|  | Unknown | 5,693 (91.9%) | 2,478 (56.4%) |  |
| Synchronous CRCLM | | 4,465 (72.1%) | 2,670 (60.8%) | <0.001 |
| Primary tumor resection | | 5,108 (82.%) | 3,071 (70.0%) | <0.001 |
| Systemic chemotherapy | | 3,730 (60.2%) | 2,891 (65.9%) | <0.001 |
| Hospital Medical School Affiliation | | 2,902 (52.8%) | 2,142 (54.8%) | 0.018 |
| Hospital CoC accreditation | | 2,852 (51.8%) | 2,286 (58.5%) | <0.001 |
| Hospital NCI designation | | 426 (7.8%) | 501 (12.9%) | <0.001 |
| **Abbreviations**: COC, Commission on Cancer; CRCLM, Colorectal cancer liver metastasis; NCI, National Cancer Institute; RUCA, Rural urban community area | | | | |
