# Supplementary figures and images for: Estimating the Survival Impact of Curative-Intent Liver Therapies for Colorectal Cancer Liver Metastases
Source: Ann Surg Oncol. 2025 May 22;32(9):6263–71. doi: 10.1245/s10434-025-17486-4 (PMC12317857; doi:10.1245/s10434-025-17486-4)

Supplemental Figure 1

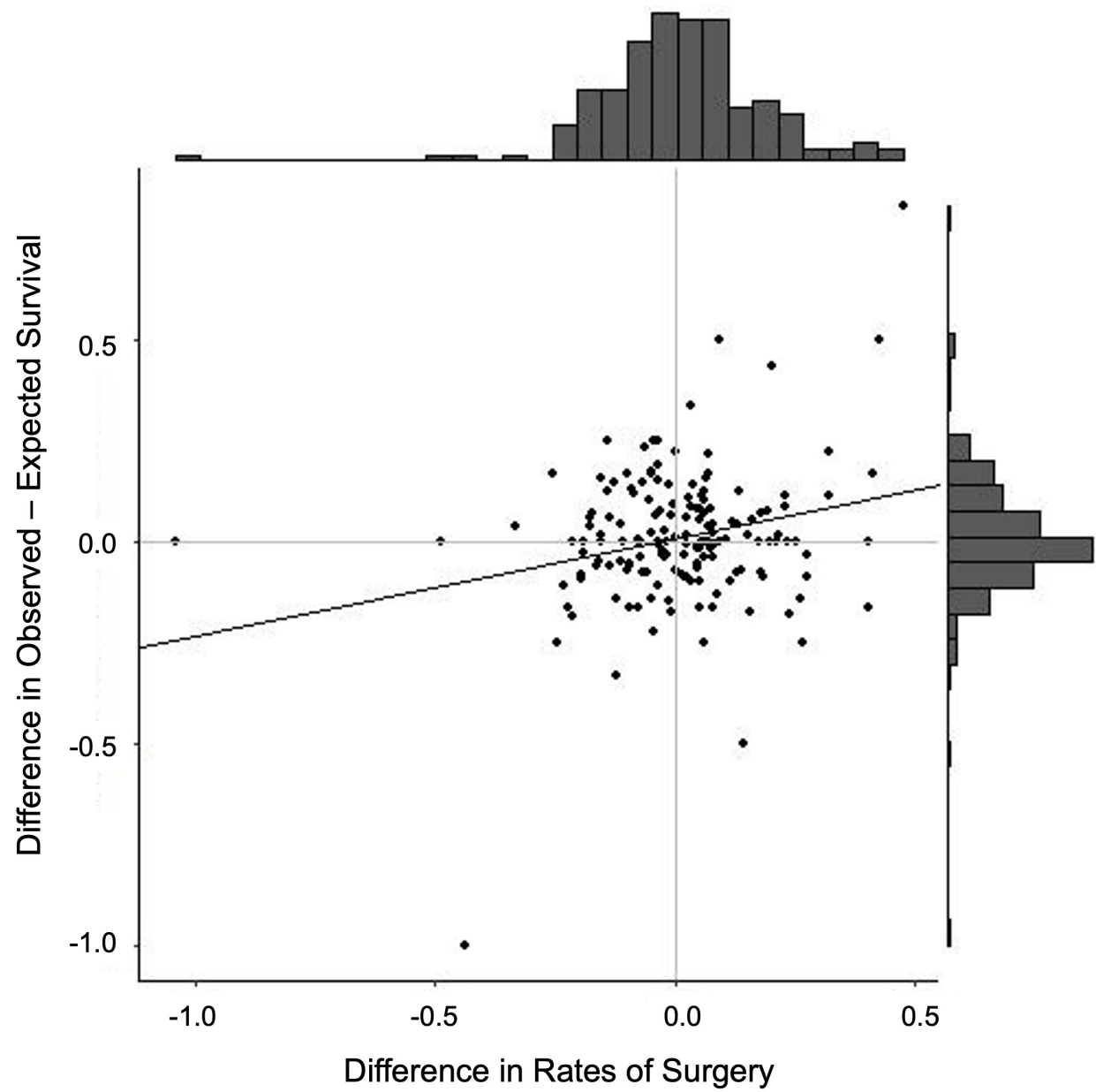

Supplemental Figure 2

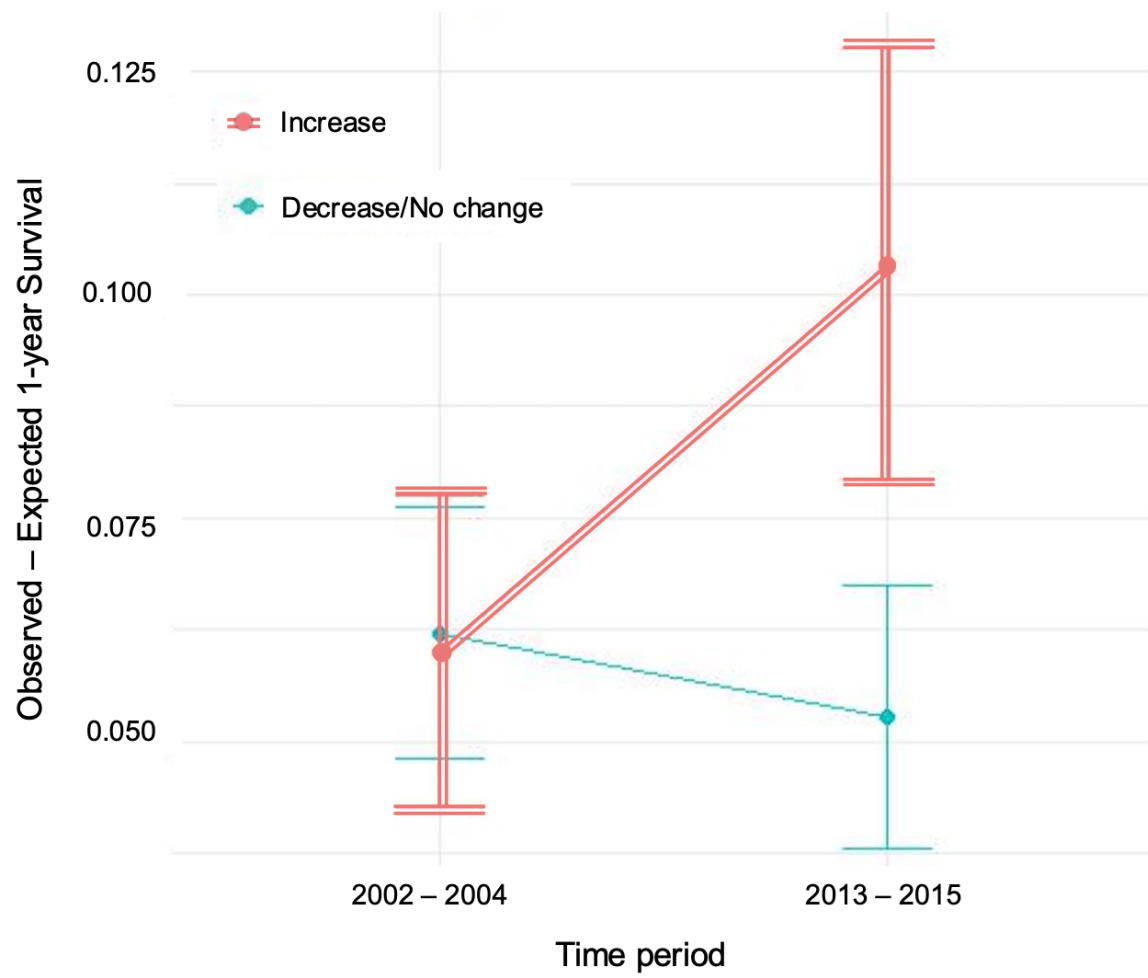

Supplemental Figure 3

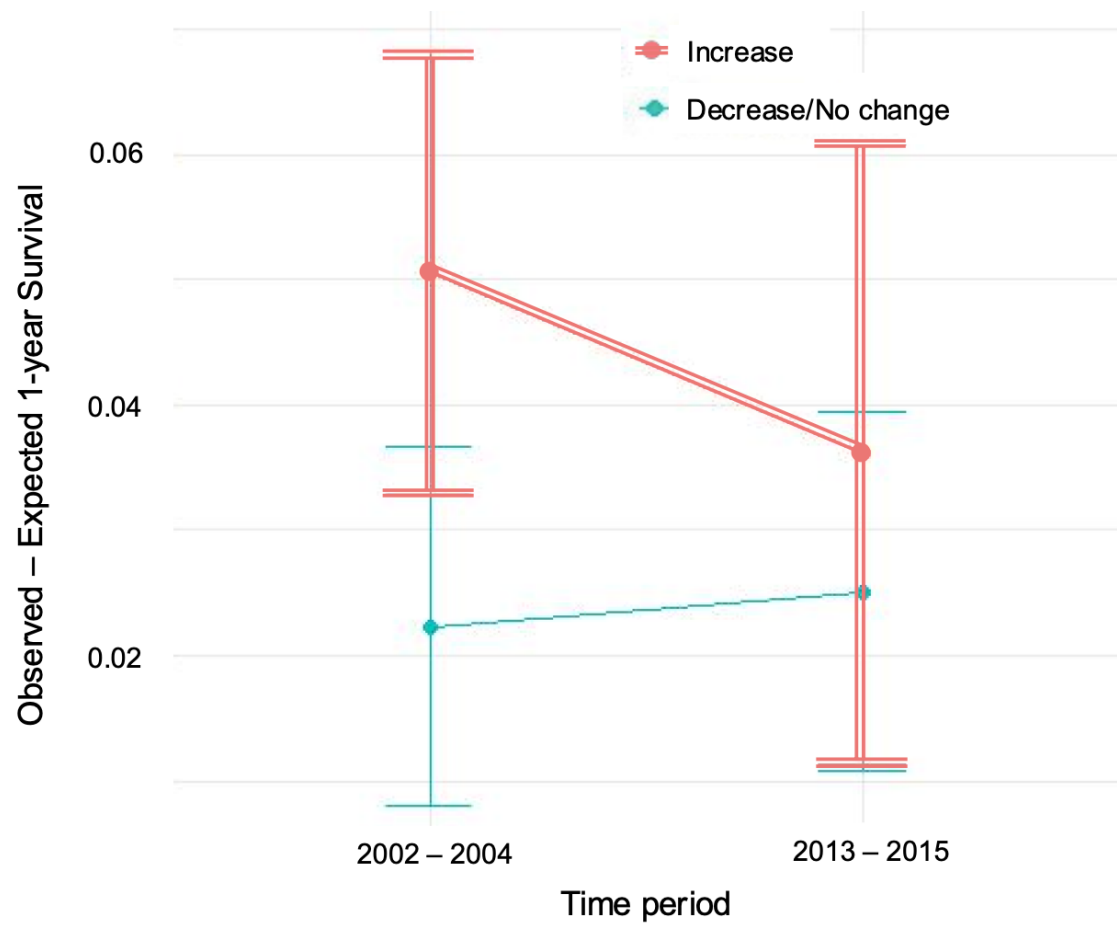

Supplement: Supplementary file 2 — Supplemental Figure 1. Graphical representation of the area-level analysis. Each point on the scatterplot represents an HSA. The x-axis measures the change in rates of surgery between the two time periods (Era 1: 2002-2004; Era 2: 2013-15). The y-axis measures the change in the observed – expected 1-year survival between the two time periods. The fitted regression line shows the relationship between these measures. The marginal histograms show the distribution of the changes. Supplemental Figure 2. Changes in observed vs. expected survival rates across eras, stratified by health service areas where the rate of curative-intent liver therapy increased (red double line) or decreased/remained unchanged (blue single line) for the subset of patients who received any cancer-directed therapy (e.g., chemotherapy, curative-intent liver therapy or both). Supplemental Figure 3. Changes in observed vs. expected survival rates across eras, stratified by health service areas where the rate of curative-intent liver therapy increased (red double line) or decreased/remained unchanged (blue single line) for the subset of patients who did not receive curative-intent liver therapy (PDF 1076 KB) [file 10434_2025_17486_MOESM2_ESM.pdf]
